# Effectiveness of vestibular rehabilitation therapy and yoga in the management of chronic peripheral vertigo: A randomized controlled trial

**DOI:** 10.12688/f1000research.147142.1

**Published:** 2024-06-05

**Authors:** K. Vaishali, Chandra Prasad Kishore, Chandra Prasasd Rao Sampath, Jeganathan P. S.

**Affiliations:** 1 Department of Physiotherapy, Manipal College of Health Professions, Manipal Academy of Higher Education, Manipal, Karnataka, 576104, India; 2 ENT and Head and Neck Surgery, Medeor Hospitals Limited, Dubai, United Arab Emirates; 3 Manipal Hospitals, Bangalore, Karnataka, India; 4 Physiology, A J Hospital and Research Center, Mangalore, Karnataka, 575004, India

**Keywords:** Chronic Vertigo, Dizziness, Dizziness Handicap Inventory, Physiotherapy, Vestibular Rehabilitation, Vertigo, Yoga

## Abstract

**Background:**

The purpose of the study was to compare the effectiveness of yoga as a form of Vestibular Rehabilitation (VR) to standard VR for managing patients with symptoms such as dizziness, disequilibrium and gait instability.

**Methods:**

150 participants based on 18-point difference in the DHI score were randomly assigned to group 1- Yoga, group 2- VR and group 3- control group using block randomization. The intervention was provided for 12 weeks. The participants were assessed for Dizziness Handicap Inventory (DHI) at baseline, 4 ^th^, 8 ^th^ and 12 ^th^ week.

**Results:**

The mean DHI for group 1(41.12±7.13) group 2 (42.96±10.54) group 3 (50.84±10.78), p<0.001 decreased significantly in group 1 and 2 when compared to baseline. There was no statistically significant difference in overall Dizziness Handicap Inventory (DHI) scores between the Yoga and Physiotherapy groups after one month; however, both groups resulted in a significant decrease in scores when compared to the control group. Similarly, by the end of the second and third months, there was no significant distinction between the Yoga and Physiotherapy groups, even though both had a considerable decrease in DHI scores when compared to the control group. Furthermore, an examination of the functional, emotional, and physical components of DHI demonstrated persistent trends of significant improvement in both the Yoga and Physiotherapy groups as compared to the control group over a three-month period.

**Conclusions:**

In addition to VR, Yoga and medications administered concurrently can provide effective therapeutic effects. Yoga has an advantage over VR since it offers a customized cure for giddiness in addition to symptom relief. Yoga might be a great alternative to the conventional VR because along with enhancing overall body relaxation, it is affordable and is easy to learn.

## Introduction

Individuals suffering from vestibular diseases usually experience dizziness and difficulty with vision, balance, and mobility. ^1^ Unilateral and peripheral vestibular diseases (UPVD) affect only one side of the vestibular system (unilateral) and only the portion of the system located outside the brain (peripheral, which is part of the inner ear). ^1^^,^^2^

Even though dizziness is a frequent complaint, clinicians typically have trouble diagnosing and treating it. They invest significantly on diagnostics but receive little in the way of conclusive results. ^2^^,^^3^ Vertigo is the delusional feeling of motion that occurs while a person is motionless and can occur within the body or in the surroundings. It is frequently accompanied by a whirling sensation, nausea, emesis, and diaphoresis. Chronic vertigo is characterized as persistent vertigo. ^4^^–^^6^ Recurrent episodes of vertigo are a hallmark of chronic vertigo. Recurrent spontaneous vertigo is frequently brought on by benign paroxysmal positional vertigo, Ménière’s syndrome, vertebra-basilar transient ischemic episodes, and migraine headaches. True labyrinthitis (sudden onset vertigo with hearing loss) and vestibular neuronitis (sudden onset vertigo without hearing loss) are monophasic illnesses that result in long-term disability. ^4^ Treatment is symptomatic, with antivertiginous and antiemetic medications used to alleviate symptoms. However, according to numerous investigations of the treatment of vertigo, none of the conventional medications have a proven curative or preventative benefit or are suitable for long-term palliative usage. ^4^

For the treatment of these conditions, the use of vestibular rehabilitation (VR), which combines various movement-based regimens, is growing. VR includes components such as intentionally inducing symptoms to “desensitize” the vestibular system, synchronizing eye and head movements, improving balance and walking abilities, and acquiring knowledge about the illness in order to cope better or engage in healthier lifestyles. ^7^

Studies, including double-blind placebo-controlled trials, have extensively validated the use of VR in a range of diseases linked with vertigo. ^8^^–^^11^ Except in a few rare instances (like BPPV treatment), these interventions are not “powerful” in the sense that their impact is usually small yet essential. ^12^ A well-known and frequently used method of VR in chronic vertigo brought on by central compensation is the Cawthorne Cooksey exercises. ^13^^–^^17^ While consistent practice of these exercises delivers beneficial results in the long term, some patients find them tedious, leading to a lack of compliance. ^15^^–^^17^ Balance activities like Yoga, Tai Chi, and martial arts are excellent alternatives to these occasionally dull exercises because they incorporate plenty of head movement and visual stimulation. ^18^

Yoga is a popular kind of exercise that promotes a healthy lifestyle and relieves a variety of human ailments. It originated in India and has since expanded throughout the world. For thousands of years, people have practiced yoga. It is made up of old presumptions, observations, and concepts about the relationship between the mind and the body that have recently been validated by modern medicine. The health benefits of yoga have been thoroughly researched, including the Yoga Postures (Asanas), Yoga Breathing (Pranayama), and Meditation. ^6^ Yoga includes relaxation, which may be beneficial to individuals who experience anxiety due to imbalance or dizziness. Yoga also has the advantage of being accessible to people of virtually any age. Even though yoga is less expensive than personalized therapy, its efficacy has not been directly compared to that of tailored therapy. The individuals who are most suitable for them are probably those who have “graduated” from individual therapy. ^19^ The purpose of this study was to use Yoga Postures (Asanas) as part of VR by employing central compensation for peripheral vertigo. In addition to enhancing focus, concentration, and a general sense of well-being, some yoga positions may also aid with balance impairment and dizzy spells by offering VR, which may be helpful in the treatment of vertigo. The aim of this research has been to evaluate the effectiveness of yoga as a vestibular rehabilitation activity to traditional Vestibular Rehabilitation (VR).

## Methods

### Study design

We conducted an observer-blinded, randomized controlled trial comparing Yoga, VR, and Pharmacotherapy in the academic department of Otolaryngology-Head & Neck Surgery, Kasturba Medical College, MAHE, Mangalore, Karnataka, between August 2009 and September 2012. The trial was reported in accordance with the guidelines of the Consolidated Standards of Reporting Trials (CONSORT) 2010 ^20^ and is registered at the Clinical Trials Registry of India as CTRI/2019/03/017995 (Date of Registration: 08/03/2019; https://ctri.nic.in/Clinicaltrials/pmaindet2.php?EncHid=MjUxMTQ=&Enc=&userName=CTRI/2019/03/017995). This clinical trial was registered retrospectively because regulatory institutional policies related to trial registration had changed after the study was completed. In order to meet the new regulations and guarantee that the trial was carried out openly and in compliance with current standards, this retrospective registration was required. The research was carried out in compliance with the Helsinki Declaration and received approval from the Kasturba Medical College and Kasturba Hospital Institutional Ethics Committee, Mangalore, MAHE (date of approval: 04/08/2009). This study adheres to the CONSORT guidelines. ^21^ The Institutional Ethics Committee (IEC) at our institution did not have a registration number at the time of the ethical registration process. As a result, even though our study was approved, no ethical approval number was issued.

### Participants

The inclusion of 150 participants in the study was based on an 18-point difference in the DHI score with a standard deviation of 21.9. Otolaryngologist diagnosed vertigo participants in the age group of 18 to 75 years were considered eligible. It was followed by a detailed examination and investigation procedure which included Electroneurography (ENG) to rule out pathologies other than peripheral vestibular disorders. Subjects with peripheral vestibular disorders were then referred to a vertigo clinic. Those subjects who fulfilled the research criteria were included in the study. The study included patients with BPPV, Labyrinthitis, Vestibular Neuronitis, Meniere’s disease, and local trauma who had chronic peripheral vestibular vertigo for at least a year and had not been recovered by treatments. Patients experiencing vertigo caused by middle ear conditions such as effusions, perilymphatic fistula, Otosclerosis, or Mastoiditis were excluded from the study. Patients with central vertigo, basilar artery insufficiency, or chronic unexplained vertigo were also excluded from participating.

### Randomization and blinding

**Figure 1.**
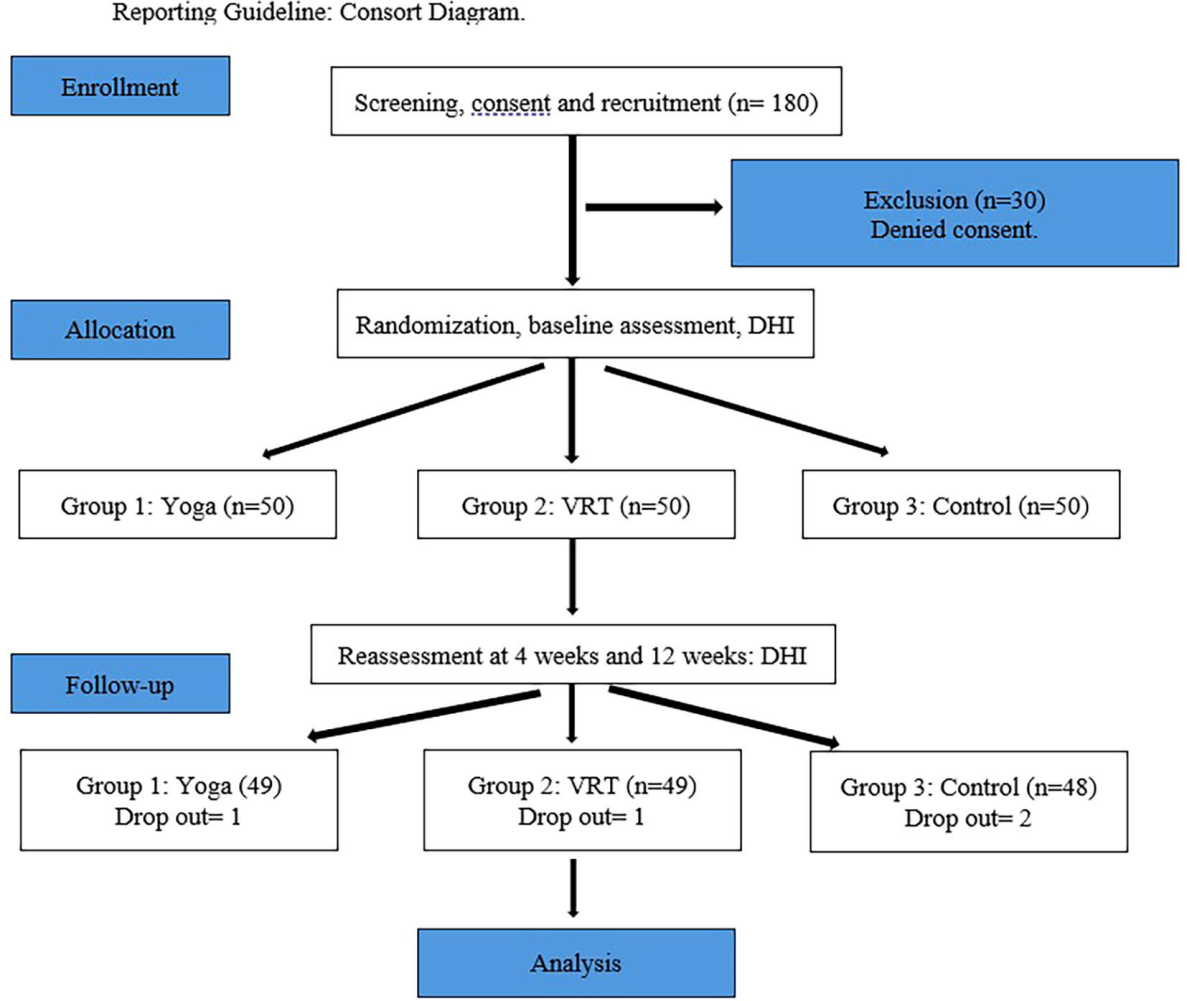
Reporting guideline: Consort diagram.

Individuals suffering from vertigo are randomized 1:1 into three groups using sealed envelopes: yoga, VR, and control. The allocation sequence was produced by a computer in order to ensure fair recruitment throughout the research. The person recruiting the participants was not given the allocation sequence, and the randomization code was kept in sealed opaque envelopes. The allocation sequence was generated by a faculty member from the Department of Physiotherapy who was not involved in the study. After obtaining written informed consent and eligibility screening, 150 participants were randomly assigned into three groups: patients treated with yoga were categorized as Group I (n=50), physiotherapy as Group II (n=50), and those with medication as Group III (n=50) through block randomization (10 blocks of 15 subjects in each block) ( Figure 1). Patients in Group I (Yoga) were subjected to a series of 20 asanas by the physiotherapist who was also a certified yoga trainer. Patients in Group II (Physiotherapy) were subjected to a series of established central compensatory exercises by the same physiotherapist. Patients in group III (Control) were treated for giddiness by medication alone by the Otolaryngologist. The outcome measures were assessed by another assessor who was not a part of the study intervention. The statistician who performed the data analysis was blinded to group allocation ( Figure 1).

### Data collection procedure

At baseline, participants were assessed for baseline information and DHI. Demographic data was obtained from the subjects. The blinded observer evaluated baseline evaluation for Dizziness Handicap Inventory (DHI). Reassessments of the outcome measures were conducted by the blinded observer 4 weeks, 8 weeks and 12 weeks. Data obtained during the research was kept absolutely confidential and was housed in a secure research server at MAHE’s Department of Physiotherapy, to which only the project investigators had access. Only de-identified data was evaluated after each participant was assigned an individual trial identification number. On the secure research server, the identifying key was kept in a separate file. The data set was only accessible to the project investigators.

### Outcomes measures

The Dizziness Handicap Inventory (DHI) was used to assess the key outcome measures at baseline, 4 weeks, 8 weeks, and 12 weeks. DHI is made up of 25 self-perceived handicap components. A total score of 100 is generated, with higher scores indicating a greater level of self-perceived handicap. Individual scores for each of the three subscales (functional, emotional, and physical) are also computed.

### Intervention (Group 1)

The subjects in Group I were treated with 12 week course of yoga therapy. During the first week, patients attended a series of 30-45 minutes per session. Subsequently the patients were instructed to continue the prescribed yoga techniques once a day for 11 weeks at home independently. Subjects were provided with an audio cassette regarding the instructions for performing the asanas. Further written format of yoga techniques was provided to the subjects. Adherence to exercise was noted through telephone once in 15 days by the investigator. Yoga techniques employed were Svastikasana, Vajrasana, Sputavajrasana, Tadasana, Padahastasana, Trikonāsana, Parsvakonasana, Parshvottanasana, Virabhadrasana, Paschimothanasana, Pooravothanasana, Pavanamuktasana, Makarāsana, Bhujangasana, Navasana, Uttanapadasana, Ujjayi pranayama, Anuloma viloma pranayama, Trataka (concentrated gazing) and Savasana. ^13^^,^^14^

### Intervention (Group 2)

The subjects in Group II were given a 12-week course of vestibular exercise. During the first week, the patient participated in a twenty-minute supervised vestibular training program in the morning and a twenty-minute unsupervised session in the evening. VR included vestibular adaptation by inducing retinal slip, eye-head exercise, remembering target exercise, resetting of VOR gain at various head speeds, ability to use somatosensory and vestibular input for postural control, ability to use vestibular and visual input for postural control, and improvement of dynamic postural control using all sensory input. Following that, the patients were instructed to complete the specified vestibular training regimen twice a day at home for 11 weeks on their own. Subjects were provided with an audio cassette regarding the instructions for performing the exercise. Further written formate of the exercises was provided to the subjects. Adherence to exercise was noted through telephone once in 15 days by the investigator.

### Intervention (Group 3)

All the subjects in Group III were treated with medicines alone. All patients were treated with Prochlorperazine (Stemetil MD) 5-10 mg three to four times daily starting from the least dose to increasing doses that were necessary to control the symptom.

### Data analysis

The statistical analysis was carried out using Jamovi 2.2.5 software https://www.jamovi.org/. The demographic variables were computed using descriptive statistics. The outcome measure findings were described using the mean and standard deviation (SD). The Shapiro-Wilk test was used to determine the normality of the variable. The Dizziness Handicap Inventory (DHI) was analyzed using Repeated Measures ANOVA across the 3 timepoints for the 3 groups. Post-Hoc Analysis was used to assess multiple comparisons within and across groups. The Bonferroni test was used to examine multiple comparisons within and between groups. Statistical significance was determined when p<.05.

## Results

Fifty subjects were included in Group I (Yoga), fifty subjects in Group II (VRT), and fifty in group III (Control). The results were analyzed for 150 patients of the 85 (56.7%) were females 65 (43.3%) were males. In the yoga group 30 (60%) were females 20 (40%) were males in the VRT group 24 (48%) were females 26 (52%) were males and in the control group, 31 (62%) were females 19 (38%) were males respectively. The majority of the patients with giddiness were in the fourth to six decades of life. While most patients with the diagnosis of BPPV & Meniere’s were in the age group of 41-60 years, those with Labyrinthitis and Vestibular neuritis were in the age group of 21-40 years ( Table 1a). Two patients lost to follow-up in each of the three groups. Two patients did not come after the first-month follow-up for unknown reasons. Four patients missed after two months follow up of exercise session (one due to transport difficulty, three had to be shifted their locality for their occupation).

**Table 1a.** Demographic characteristics.

Characteristics	Mean ± SD
Age:	
BPPV and Meniere’s	41-60 Years
Labyrinthitis/Vestibular nuritis	21-40 Years
Mean symptom duration (months)	15.76 ± 7.67
Common peripheral vertigo:	
BPPV	48.6%
Meniere’s	35.1%
Labyrinthitis/Vestibular nuritis	13.2 %

	Males	Females
Gender	85 (56.7%)	65 (43.3%)
Group I (Yoga)	20 (40%)	30 (60%)
Group II (VRT)	26 (52%)	24 (48%)
Group III (Control)	19 (38%)	31 (62%)

BPPV was the most common cause of peripheral vertigo (48.6%), followed by Meniere’s (35.1%) and Labyrinthitis or vestibular neuritis (13.2%) ( Table 1b). ^22^ The mean symptom duration was 15.76±7.67 months (12-72 months) for 150 patients.

**Table 1b.** Age incidence.

Diagnosis	Age (Years)				
	0-20	21-40	41-60	61-80	Total
BPPV	2 (1.3%)	18 (12%)	39 (26%)	14 (9.3%)	73 (48.6%)
Menieres	2 (1.3%)	16 (10.6%)	27 (18%)	12 (8%)	57 (38%)
Labrynthitis/Vestibular neuritis	1 (0.6%)	10 (6.6%)	6 (4%)	3 (2%)	20 (13.3%)

**Figure 2.**
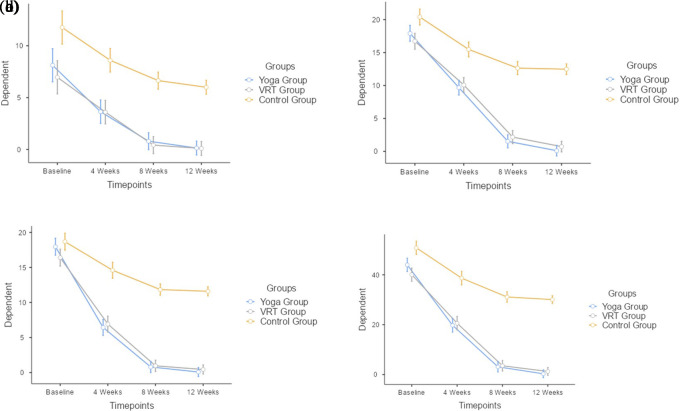
(a) Physical component of Dizziness Handicap Inventory Scale across the time points for groups 1, 2 and 3. (b) Functional component of Dizziness Handicap Inventory Scale across the time points for groups 1, 2 and 3. (c) Emotional component of Dizziness Handicap Inventory Scale across the time points for groups 1, 2 and 3. (d) Dizziness Handicap Inventory Scale total score across the time points for groups 1, 2 and 3.

The DHI-Physical at baseline mean±SD values between Yoga group (8.12±6.213), VRT group (6.96±4.802) and the control group (11.76±6.056), as shown in Table 2a, are observed to be different from each other. The DHI-Physical at 4 weeks mean±SD values between Yoga group (3.64±3.718), VRT group (3.6±3.747) and the control group (8.6±4.664), as shown in Table 3a. The DHI-Physical at 8 weeks mean±SD values between Yoga group (0.8±2.356), VRT group (0.42±0.906) and the control group (6.64±4.336), as shown in Table 4a. The DHI-Physical at 12 weeks mean±SD values between Yoga group (0.14±0.495), VRT group (0.1±0.505) and the control group (6±4.101), as shown in Table 5a. There was no statistical significance (mean difference, p value) between Yoga and VRT group (0.405, 1), statistical significance was noted between Yoga and Control group (-5.075, <.001) as well as VRT and Control group (-5.48, <.001). Based on the findings, all three groups were compared with each time point and it was found that there was a statistically significant difference (F=52, p<.001) between the 3 groups. When the groups were compared with each other, statistically significant difference (F=159.33, p<.001) were found throughout the time points. When the three groups were examined at various intervals, statistically insignificant (F=1.66, p=0.13) differences were found ( Table 2b and Figure 2a).

**Table 2a.** Physical component of Dizziness Handicap Inventory Scale across the time points for groups 1, 2 and 3.

Physical	Yoga (Mean±SD)	VRT (Mean±SD)	Control (Mean±SD)	Group [F(p)]	Timepoint [F(p)]	Time*Timepoint [F(p)]
Baseline	8.12±6.213	6.96±4.802	11.76±6.056	52 (<.001)	159.33 (<.001)	1.66 (0.13)
4 weeks	3.64±3.718	3.6±3.747	8.6±4.664			
8 weeks	0.8±2.356	0.42±0.906	6.64±4.336			
12 weeks	0.14±0.495	0.1±0.505	6±4.101			

**Table 2b.** Post-Hoc comparison between groups-DHI (P).

		Mean difference	Pbonferroni
Yoga Group	VRT Group	0.405	1
Yoga Group	Control Group	-5.075	< .001
VRT Group	Control Group	-5.48	< .001

The DHI-Functional at baseline mean±SD values between Yoga group (17.92±4.642), VRT group (16.72±3.326) and the control group (20.4±4.832), as shown in Table 3a, are observed to be different from each other. The DHI-Functional at 4 weeks mean±SD values between Yoga group (9.68±9.68), VRT group (10.08±4.198) and the control group (15.48±3.829), as shown in Table 3a. The DHI-Functional at 8 weeks mean±SD values between Yoga group (1.52±3.247), VRT group (2.16±3.203) and the control group (12.898±3.721), as shown in Table 4a. The DHI-Functional at 12 weeks mean±SD values between Yoga group (0.1±0.416), VRT group (0.72±1.97) and the control group (12.48±4.564), as shown in Table 5a. There was no statistical significance (mean difference, p value) between Yoga and VRT group (-0.115, 1), statistical significance was noted between Yoga and Control group (-7.945, <.001) as well as VRT and Control group (-7.83, <.001). Based on the findings, all three groups were compared with each time point and it was found that there was a statistically significant difference (F=156, p<.001) between the 3 groups. When the groups were compared with each other, statistically significant difference (F=641.3, p<.001) were found throughout the time points. When the three groups were examined at various intervals, statistically significant (F=32.1, p<.001) differences were found ( Table 3b and Figure 2b).

**Table 3a.** Functional component of Dizziness Handicap Inventory Scale across the time points for groups 1, 2 and 3.

Functional	Yoga (Mean±SD)	VRT (Mean±SD)	Control (Mean±SD)	Group [F(p)]	Timepoint [F(p)]	Time*Timepoint [F(p)]
Baseline	17.92±4.642	16.72±3.326	20.4±4.832	156 (<.001)	641.3 (<.001)	32.1 (<.001)
4 weeks	9.68±9.68	10.08±4.198	15.48±3.829			
8 weeks	1.52±3.247	2.16±3.203	12.898±3.721			
12 weeks	0.1±0.416	0.72±1.97	12.48±4.564			

**Table 3b.** Post-Hoc comparison between groups-DHI (F).

		Mean difference	Pbonferroni
Yoga Group	VRT Group	-0.115	1
Yoga Group	Control Group	-7.945	< .001
VRT Group	Control Group	-7.83	< .001

The DHI-Emotional at baseline mean±SD values between Yoga group (17.96±3.928), VRT group (16.4±4.295) and the control group (18.68±4.688), as shown in Table 4a, are observed to be different from each other. The DHI-Emotional at 4 weeks mean±SD values between Yoga group (6.44±3.732), VRT group (6.92±4.375) and the control group (14.6±4.086), as shown in Table 3a. The DHI-Emotional at 8 weeks mean±SD values between Yoga group (0.8±2.65), VRT group (0.96±1.818) and the control group (12.0816±3.415), as shown in Table 4a. The DHI-Emotional at 12 weeks mean±SD values between Yoga group (0.08±0.566), VRT group (0.44±1.527) and the control group (11.6±3.681), as shown in Table 5a. There was no statistical significance (mean difference, p value) between Yoga and VRT group (0.14, 1), statistical significance was noted between Yoga and Control group (-7.86, <.001) as well as VRT and Control group (-8, <.001). Based on the findings, all three groups were compared with each time point and it was found that there was a statistically significant difference (F=189, p<.001) between the 3 groups. When the groups were compared with each other, statistically significant difference (F=674.2, p<.001) were found throughout the time points. When the three groups were examined at various intervals, statistically significant (F=40.1, p<.001) differences were found ( Table 4b and Figure 2c).

**Table 4a.** Emotional component of Dizziness Handicap Inventory Scale across the time points for groups 1, 2 and 3.

Emotional	Yoga (Mean±SD)	VRT (Mean±SD)	Control (Mean±SD)	Group [F(p)]	Timepoint [F(p)]	Time*Timepoint [F(p)]
Baseline	17.96±3.928	16.4±4.295	18.68±4.688	189 (<.001)	674.2 (<.001)	40.1 (<.001)
4 weeks	6.44±3.732	6.92±4.375	14.6±4.086			
8 weeks	0.8±2.65	0.96±1.818	12.0816±3.415			
12 weeks	0.08±0.566	0.44±1.527	11.6±3.681			

**Table 4b.** Post-Hoc comparison between groups-DHI (E).

		Mean difference	Pbonferroni
Yoga Group	VRT Group	0.14	1
Yoga Group	Control Group	-7.86	< .001
VRT Group	Control Group	-8	< .001

The DHI-Total at baseline mean±SD values between Yoga group (44±10.482), VRT group (40.08±6.797) and the control group (50.84±10.782), as shown in Table 5a, are observed to be different from each other. The DHI-Total at 4 weeks mean±SD values between Yoga group (19.76±9.812), VRT group (20.6±10.461) and the control group (38.68±8.977), as shown in Table 3a. The DHI-Total at 8 weeks mean±SD values between Yoga group (3.12±7.774), VRT group (3.54±5.011) and the control group (31.7551±7.965), as shown in Table 4a. The DHI-Total at 12 weeks mean±SD values between Yoga group (0.32±1.096), VRT group (1.26±3.312) and the control group (30.08±9.026), as shown in Table 5a. There was no statistical significance (mean difference, p value) between Yoga and VRT group (0.43, 1), statistical significance was noted between Yoga and Control group (-20.88, <.001) as well as VRT and Control group (-21.31, <.001). Based on the findings, all three groups were compared with each time point and it was found that there was a statistically significant difference (F=245, p<.001) between the 3 groups. When the groups were compared with each other, statistically significant difference (F=743.9, p<.001) were found throughout the time points. When the three groups were examined at various intervals, statistically significant (F=30.9, p<.001) differences were found ( Table 5b and Figure 2d).

**Table 5a.** Dizziness Handicap Inventory Scale total score across the time points for groups 1, 2 and 3.

Total score	Yoga (Mean±SD)	VRT (Mean±SD)	Control (Mean±SD)	Group [F(p)]	Timepoint [F(p)]	Time*Timepoint [F(p)]
Baseline	44±10.482	40.08±6.797	50.84±10.782	245 (<.001)	743.9 (<.001)	30.9 (<.001)
4 weeks	19.76±9.812	20.6±10.461	38.68±8.977			
8 weeks	3.12±7.774	3.54±5.011	31.7551±7.965			
12 weeks	0.32±1.096	1.26±3.312	30.08±9.026			

**Table 5b.** Post-Hoc comparison between groups-DHI (T).

		Mean difference	Pbonferroni
Yoga Group	VRT Group	0.43	1
Yoga Group	Control Group	-20.88	< .001
VRT Group	Control Group	-21.31	< .001

## Discussion

This study evaluated the effectiveness of yoga as a vestibular rehabilitation strategy to standard Vestibular Rehabilitation (VR) in treating persistent peripheral vertigo. Over time, the utility of vestibular exercises for managing patients with chronic symptoms of positional vertigo and disequilibrium has been established. A customized vestibular rehabilitation treatment (VRT) program was developed to match the unique demands of each individual, while taking into account a greater understanding of the vestibular system’s function and adaptation. VRT requires active patient participation and can be delivered through supervised outpatient workouts or home exercise regimens. ^9^ Vestibular exercises are overseen and progressed by therapists, and suitable outcome measures are used to assess the patient’s progress. A VRT program typically lasts between four and ten weeks.

There is strong evidence that vestibular rehabilitation is both safe and effective in the treatment of unilateral peripheral vestibular impairment. ^10^ Vestibular rehabilitation therapy (VRT) has shown significant benefit in individuals with a stable unilateral peripheral vestibular loss and partial central compensatory. Symptoms such as skew deviation, nystagmus, vertigo, oscillopsia, disequilibrium, nausea, and vomiting often disappear after an acute vestibular dysfunction, such as vestibular neuritis, as the central nervous system (CNS) achieves acute compensation during the first 24 to 72 hours. The cerebellum influences this compensation, which requires releasing cerebellar inhibition to establish symmetrical tonic firing rates in second-order neurons originating in the vestibular nuclei. ^7^ While some patients recover quickly and spontaneously, individuals who have partial compensation or decompensation of the CNS to the vestibular system after the acute lesion may continue to have motion-induced vertigo, oscillopsia, and disequilibrium. ^11^ Individuals in such instances may benefit from a personalized term of VRT guided by a skilled vestibular therapist. ^3^

Horak and colleagues found that after a 6-week intervention period involving treatment groups (vestibular rehabilitation therapy or VRT, general conditioning exercise, and medication), all three approaches led to a reduction in dizziness symptoms in individuals with chronic unilateral vestibular loss (UVL). However, only VRT demonstrated a significant increase in balance. ^12^ According to Mira E’s review, improving the quality of life for individuals with peripheral vestibular diseases is a main goal of therapeutic therapies. ^11^ Furthermore, Gottshall and colleagues determined that vestibular therapy in individuals with Meniere’s illness resulted in considerable improvements in balance function, as measured by objective and self-reported tests. ^13^

Persistent dizziness is commonly associated with considerable handicap, impairment, and psychological distress, and it frequently causes secondary concerns such as anxiety, hyperventilation, and neck pain as a result of keeping a stiff head position in order to avoid triggering head movements. ^8^ Chronic dizziness requires a multimodal approach, with rehabilitation and simple counseling being required for all patients. ^14^^,^^15^ Although exercises produce favorable outcomes when practiced consistently over time, some individuals may find them monotonous, leading to compliance concerns. Engaging in balance exercises such as Yoga, Tai Chi, martial arts, or games involving significant head movement and visual stimulation is another option worth investigating. Both Tai Chi and Yoga contain relaxing elements that might be beneficial, particularly for people with anxiety. Although their effectiveness has not been directly compared, these activities are often more cost-effective than individualized therapy. They are most likely appropriate for people who have finished personalized therapy. ^2^

Yoga, which originated in India and is now extensively practiced worldwide, has become a popular type of exercise that provides a holistic approach to a healthy lifestyle as well as treatment from a variety of human ailments. Yoga, which has been practiced for over 5,000 years, incorporates breathing techniques, physical postures, and meditation. While yoga began as a spiritual practice inside Hinduism, a subset of the discipline known as Asana has grown in popularity in the Western world primarily as a physical fitness program. Yoga is frequently isolated from Hinduism or spirituality in Western contexts, serving only as a technique of preserving physical fitness and health. Clinical investigations are increasingly being conducted to determine the extent to which yoga helps psychological and emotional well-being. A PubMed search for “yoga and depression” yields 25 clinical trials concentrating on the association between yoga and emotional and psychological health published during 2007, 2008, and the first quarter of 2009. Moreover, three review articles and three systematic reviews were published over this time period to study the effects of various combinations of yoga, meditation, and yogic breathing on mental health.

Yoga incorporates ancient theories and principles concerning the relationship between the mind and body, which modern medicine is now proving through extensive research. Extensive research has been done to investigate the health benefits of many parts of yoga, such as yoga postures (asanas), yoga breathing (pranayama), and meditation. The material on the benefits of Yoga Poses is divided into three categories: physiological, psychological, and scientific comparisons to the benefits of regular exercise. ^16^ Yoga’s physiological benefits include the establishment of stable autonomic nervous system equilibrium, a drop in pulse rate, a reduction in respiratory rate, improved eye-hand coordination, a reduction in reaction time, and good effects on posture and balance. Yoga is connected with increased somatic and kinesthetic awareness, mood enhancement, enhanced subjective well-being, improved concentration, better memory, heightened attention, and an overall rise in well-being. These findings have been objectively examined and compared to the advantages of regular exercise. ^17^^,^^18^

The purpose of this study was to investigate the use of Yoga Postures (Asanas) as a component of vestibular therapy for central compensation in cases with peripheral vertigo. The hypothesis proposed that various Yoga postures could not only aid in vestibular rehabilitation but also reduce dizzy episodes while improving focus, concentration, and overall well-being, hence assisting in vertigo recovery. Another advantage of yoga is that it is appropriate for individuals of all ages. The study aimed to assess the efficacy of yoga vs physiotherapy (VRT), using yoga poses chosen to promote optimal movement of the head, balance, and body for vestibular rehabilitation and central compensation. In an observer-blinded randomized controlled study, the Dizziness Handicap Inventory (DHI) was used. The results showed that, as shown in the tables, yoga was comparable to VRT in all of the evaluated criteria.

## Conclusion

The advantage of yoga over physiotherapy or VRT is that it provides a wholesome and customized cure for giddiness. That means, apart from providing symptomatic relief of giddiness by vestibular rehabilitation, yoga also brings in additional proven benefits like general body relaxation, increased blood oxygenation, detoxification, and an overall sense of well-being. Being cheap and easy to learn, yoga could be an excellent substitute for the traditional VRT.

## Ethics and consent

The trial was registered at the Clinical Trials Registry of India as CTRI/2019/03/017995 (Date of Registration: 08/03/2019; https://ctri.nic.in/Clinicaltrials/pmaindet2.php?EncHid=MjUxMTQ=&Enc=&userName=CTRI/2019/03/017995). This clinical trial was registered retrospectively because regulatory institutional policies related to trial registration had changed after the study was completed. In order to meet the new regulations and guarantee that the trial was carried out openly and in compliance with current standards, this retrospective registration was required. The research was carried out in compliance with the Helsinki Declaration and received approval from the Kasturba Medical College and Kasturba Hospital Institutional Ethics Committee, Mangalore, MAHE (date of approval: 04/08/2009). This study adheres to the CONSORT guidelines. ^21^ The Institutional Ethics Committee (IEC) at our institution did not have a registration number at the time of the ethical registration process. As a result, even though our study was approved, no ethical approval number was issued. The participants provided their written informed consent to participate in the study. We confirm that we obtained written informed consent from all participants involved in our study to publish the study results and that all personal information will be kept confidential.

## Data Availability

Harvard Dataverse: Replication Data for: Datasheet. https://doi.org/10.7910/DVN/RUCLSQ. ^22^ Harvard Dataverse: CONSORT checklist for ‘Effectiveness of vestibular rehabilitation therapy and yoga in the management of chronic peripheral vertigo: A randomized controlled trial’. https://doi.org/10.7910/DVN/QRMGP3. ^21^ Data are available under the terms of the Creative Commons Zero “No rights reserved” data waiver (CC0 1.0 Public domain dedication).
