# Supplementary material for: Effectiveness of vestibular rehabilitation therapy and yoga in the management of chronic peripheral vertigo: A randomized controlled trial
Source: F1000Res. 2024 Jun 5;13:578. [Version 1] doi: 10.12688/f1000research.147142.1 (PMC11406116; doi:10.12688/f1000research.147142.1)
Supplement: Peer review history

# Reviewer report: Reviewer response for version 1

**DOI:** 10.5256/f1000research.161297.r288113

**Published:** 2024-09-16

**Authors:** Jaya Shanker Tedla

**Affiliations:** 1 Department of Medical Rehabilitation Sciences, College of Applied Medical Sciences, King Khalid University, Abha, Aseer Province, Saudi Arabia

An interdisciplinary study using Yoga for vestibular rehabilitation. The authors nicely executed it. However, answer a few of my comments so that readers will understand it more. Thank you

 1. We need to discuss the reliability and clinometric properties of the outcome measures you used in the outcome measures section.

 2. Kindly mention clearly in the manuscript that these supervised sessions are part of familiarization and precisely what is taught in familiarization sessions should

 be implemented in unsupervised sessions.

 3. The telephone call details should be described thoroughly in the methods section. Specify the motivational phrases used and explain if they were consistent for all

 participants or varied depending on their level of motivation. Additionally, specify the criteria used to measure and categorize the motivation levels.

 4. How was adherence noted? Any tool used and at what intervals?

 5. Highlighting its contribution to the field in the discussion would be beneficial.

Is the work clearly and accurately presented and does it cite the current literature?

Yes

If applicable, is the statistical analysis and its interpretation appropriate?

Yes

Are all the source data underlying the results available to ensure full reproducibility?

Yes

Is the study design appropriate and is the work technically sound?

Yes

Are the conclusions drawn adequately supported by the results?

Yes

Are sufficient details of methods and analysis provided to allow replication by others?

Yes

Reviewer Expertise:

Physical Therapy and Rehabilitation

I confirm that I have read this submission and believe that I have an appropriate level of expertise to confirm that it is of an acceptable scientific standard.

# Reviewer report: Reviewer response for version 1

**DOI:** 10.5256/f1000research.161297.r298663

**Published:** 2024-07-22

**Authors:** Snehal Subrat Samal

**Affiliations:** 1 Department of Neuro Physiotherapy, Datta Meghe Institute of Higher Education & Research, Wardha, Maharashtra, India

The article is impeccably crafted and exhibits a high level of written proficiency. The elucidation provided for each of the three distinct groups is thorough and comprehensive, demonstrating a commendable attention to detail and accuracy in explication. One notable aspect deserving of further scrutiny pertains to the temporal parameters governing the administration of treatments across the groups. It is evident that the duration of treatment varies among the groups, posing a potential factor that may influence the outcomes observed. In addition, an intriguing proposition emerges from the author's perspective, suggesting the incorporation of audio cassette supplements alongside the medication regimen within the third group. This proposal warrants careful consideration and warrants a more in-depth exploration to assess its potential impact on treatment efficacy. Furthermore, the proposal to utilize the balance scale as an alternative outcome measure is a commendable suggestion, especially in light of the limitations associated with the exclusive reliance on the Dizziness Handicap Inventory (DHI) as a measure of Activities of Daily Living (ADL). The author's rationale for this recommendation is cogently articulated, underscoring the importance of incorporating a diverse array of metrics to capture the multifaceted nature of treatment outcomes. Lastly, the statistical analyses presented in the article are characterized by a high degree of clarity and precision, facilitating a comprehensive understanding of the data and findings.

 All the best., I appreciate your work and study

Is the work clearly and accurately presented and does it cite the current literature?

Yes

If applicable, is the statistical analysis and its interpretation appropriate?

Yes

Are all the source data underlying the results available to ensure full reproducibility?

Yes

Is the study design appropriate and is the work technically sound?

Yes

Are the conclusions drawn adequately supported by the results?

Yes

Are sufficient details of methods and analysis provided to allow replication by others?

Yes

Reviewer Expertise:

Neurology and Neuro-rehabilitation

I confirm that I have read this submission and believe that I have an appropriate level of expertise to confirm that it is of an acceptable scientific standard.
